# Heterogeneity of variance components for preweaning growth in Romane sheep due to the number of lambs reared

**DOI:** 10.1186/1297-9686-43-32

**Published:** 2011-09-07

**Authors:** Ingrid David, Frédéric Bouvier, Dominique François, Jean-Paul Poivey, Laurence Tiphine

**Affiliations:** 1 INRA UR 631, Station d'Amélioration Génétique des Animaux, 31320 Castanet-Tolosan, France; 2 INRA UE 0332, Domaine de la Sapinière, 18390 Osmoy, France; 3 CIRAD UMR 112, SELMET, 34398 Montpellier, France; 4 Institut de l'Elevage, 75012 Paris, France

## Abstract

**Background:**

The pre-weaning growth rate of lambs, an important component of meat market production, is affected by maternal and direct genetic effects. The French genetic evaluation model takes into account the number of lambs suckled by applying a multiplicative factor (1 for a lamb reared as a single, 0.7 for twin-reared lambs) to the maternal genetic effect, in addition to including the birth*rearing type combination as a fixed effect, which acts on the mean. However, little evidence has been provided to justify the use of this multiplicative model. The two main objectives of the present study were to determine, by comparing models of analysis, 1) whether pre-weaning growth is the same trait in single- and twin-reared lambs and 2) whether the multiplicative coefficient represents a good approach for taking this possible difference into account.

**Methods:**

Data on the pre-weaning growth rate, defined as the average daily gain from birth to 45 days of age on 29,612 Romane lambs born between 1987 and 2009 at the experimental farm of La Sapinière (INRA-France) were used to compare eight models that account for the number of lambs per dam reared in various ways. Models were compared using the Akaike information criteria.

**Results:**

The model that best fitted the data assumed that 1) direct (maternal) effects correspond to the same trait regardless of the number of lambs reared, 2) the permanent environmental effects and variances associated with the dam depend on the number of lambs reared and 3) the residual variance depends on the number of lambs reared. Even though this model fitted the data better than a model that included a multiplicative coefficient, little difference was found between EBV from the different models (the correlation between EBV varied from 0.979 to 0.999).

**Conclusions:**

Based on experimental data, the current genetic evaluation model can be improved to better take into account the number of lambs reared. Thus, it would be of interest to evaluate this model on field data and update the genetic evaluation model based on the results obtained.

## Background

The total weight of lambs weaned per ewe is an important component of meat market production and is a function of litter size, lamb survival and lamb growth. Pre-weaning growth is a complex phenotype that is influenced by two distinct components: direct and maternal effects. The maternal effect is a strictly environmental effect on the offspring [1]; it arises from the mother's ability to produce the milk needed for growth and her maternal behaviour. The direct component corresponds to the suckling behaviour and growth ability of the young. It has been shown that these two components are heritable in sheep (as reviewed by Safari et al. [2]). The pre-weaning growth of lambs is highly dependent on the number of lambs born and suckled [3]. The number of suckling lambs modifies both the mother's milk production [4,5] and the suckling/competition behaviour of the young [6-8]. Based on the work of Ricordeau and Boccard [9], the French genetic evaluation model for pre-weaning growth [10] accounts for this effect by applying a multiplicative factor (*α*) to the maternal genetic effect (*α *= 1, 0.7 and 0.5 for one, two and more than two suckling lambs, respectively), in addition to including the birth*rearing type combination as a fixed effect, which acts on the mean. However, to date, no other argument justifying the use of this multiplicative model has been reported. Furthermore, the model seems to suffer some drawbacks since it has been reported from the field that the maternal EBV of ewes having previously reared single-suckling lambs decreases very much if they rear two or more lambs in a subsequent year.

Consequently, the aim of the present study was to determine 1) whether pre-weaning growth is the same trait in single- and twin-reared lambs; i.e. to determine whether the number of lambs suckling affects the variance components that act on pre-weaning growth, 2) whether applying the multiplicative coefficient represents an appropriate solution to account for such heterogeneity, and 3) whether, when the multiplicative coefficient is applied, the maternal EBV of ewes having previously reared single-suckling lambs decreases markedly if they rear two lambs in a subsequent year. To address these objectives, we compared eight models that allowed for heterogeneity of the various variance components for the average daily gain from 0 to 45 days of age in Romane sheep as a function of the number of lambs reared.

## Methods

### Data

**Figure 1:**
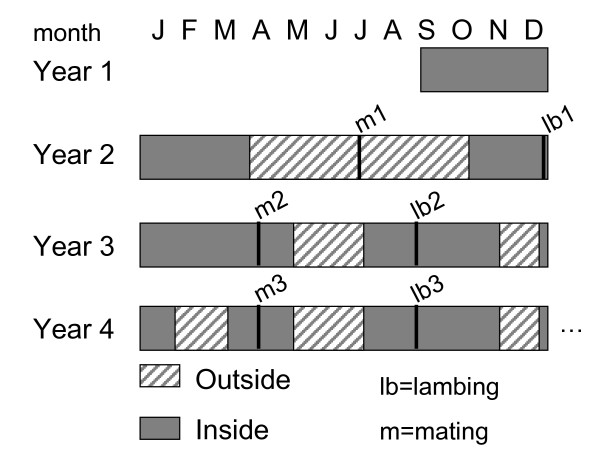
**Ewe management schemes**.

Data from Romane lambs born between 1987 and 2009 at the experimental farm of La Sapinière (INRA-France) were used in this study. This experimental population is the nucleus flock of the composite sheep strain INRA401 [11]. Only data from lambs reared as a single or twins were retained for analysis (29,612 observations, 18% reared as singles, 82% as twins). All animals were bred in the same system. During the 1987-2009 period, ewes were managed under two schemes. The management scheme used during the first part of the period is described in detail in [12]; briefly, ewes were first exposed to rams in April at 16 ± 1 months of age. Ewes that lambed in September were mated again in October at 22 ± 1 months of age. Then, for subsequent lambings, ewes were mated once a year in July-August. No lambs were retained as replacements from the first two lambings of a ewe. During the second part of the 1987-2009 period, ewes were managed under the following scheme (Figure 1): they were first exposed to rams in July at 10 ± 1 months of age. From April to September, the ewes were kept outside and then lambed indoors in December. No lambs from the first lambing were retained as replacements. The ewes were then mated once a year in April and lambed in September. These adult ewes were on pasture from mid-May to mid-July, from November to December and from February to April. Lambs were reared with their mothers from birth to weaning (60 days).

**Figure 2:**
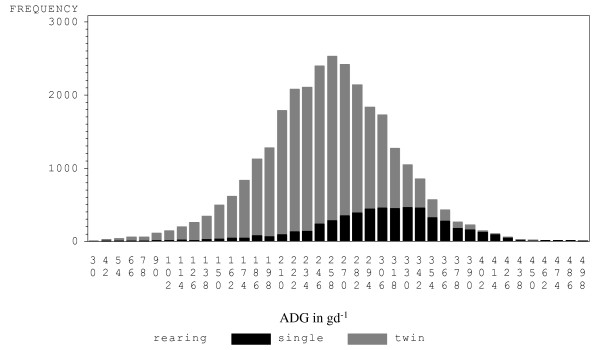
**Distribution of pre-weaning ADG (g.d^-1^) for single- and twin-reared lambs**.

Lambs were weighed at birth and at 45 days of age (on average 44.5 days (± 4.3) for single- and 44.8 days (± 3.7) for twin-reared lambs) using a standardized method (i.e. same animal restraint method, same weight scale). Resulting weights were used to calculate the average daily gain (ADG) between birth and 45 days. The average ADG was 254.9 g.d^-1 ^(± 62.1) for all lambs, 304.3 g.d^-1 ^(± 62.7) for single-reared lambs and 243.7 g.d^-1 ^(± 56.2) for twin-reared lambs. The distribution of ADG is shown in Figure 2. Pedigree information was established for 33,304 animals with minimal sire misidentification. Data are summarized in Table 1.

**Table 1:** Data description

	N	Mean (std) of number of records^1^
Lambs	29,612	
Single-reared lambs	5,479	
Twin-reared lambs	24,133	

Animals in the pedigree	33,304	-

Dam with records		
*all*	6,379	4.6 (3.2)
*rearing single lambs*	3,815	1.5 (0.9)
*rearing twins*	5,811	4.4 (3.0)

Sires of lambs with records		
*all*	683	33.2 (21.5)
*Single-reared *	640	6.1 (4.9)
*Twin-reared *	681	29.5 (19.2)

Maternal grand sires of lambs with records		
*all*	723	43.0 (32.1)
*Single-reared *	675	8.6 (7.3)
*Twin-reared *	711	35.5 (26.3)

Litters	18,269	1.6 (0.49)

^1^mean and standard deviation of number of ADG records per animal. For instance, the mean total number of lambs weighted per females rearing single is 1.5.

### Model comparison

Data were analyzed using eight distinct models which were all sub-models of the following "global" model:

$$\left\{\begin{array}{c} Y_{1}=X_{1}\beta_{1}+Z_{d1}d_{1}+\alpha_{1}*Z_{m1}m_{1}+W_{1}p_{1}+M_{1}l_{1}+\varepsilon_{1}\\ Y_{2}=X_{2}\beta_{2}+Z_{d2}d_{2}+\alpha_{2}*Z_{m2}m_{2}+W_{2}p_{2}+M_{2}l_{2}+\varepsilon_{2} \end{array}\right)$$

where subscripts 1 and 2 refer to single- and twin-reared lambs, respectively; ***Y_i _***is the vector of measured ADG for single- (i = 1) or twin-reared (i = 2) lambs; ***β_i _***is the vector of fixed effects; ***d_i _***is the vector of direct genetic effects; ***m_i _***is the vector of maternal genetic effects; ***p_i _***is the vector of permanent environmental effects for the dam; ***l_i _***is the vector of litter effects; ***ε_i _***is the vector of residuals; ***X_i_***, ***Z_di_***, ***Z_mi_***, ***W_i_***, ***M_i _***are the corresponding known incidence matrices. All random effects were distributed as centered normal distributions with variance covariance matrices equal to $A⊗\left[\begin{array}{cccc} \sigma_{d1}^{2} & \sigma_{d1d2}^{} & \sigma_{d1m1}^{} & \sigma_{d1m2}^{}\\ & \sigma_{d2}^{2} & \sigma_{d2m1}^{} & \sigma_{d2m2}^{}\\ sym & & \sigma_{m1}^{2} & \sigma_{m1m2}^{}\\ & & & \sigma_{m2}^{2} \end{array}\right]$ for the genetic effects, where ***A ***is the relationship matrix, $I_{p}⊗\left[\begin{array}{cc} \sigma_{p1}^{2} & \sigma_{p}^{}\\ \sigma_{p}^{} & \sigma_{p2}^{2} \end{array}\right]$ for the permanent effects, $\left[\begin{array}{cc} I_{l1}⊗\sigma_{l1}^{2} & 0\\ 0 & I_{l2}⊗\sigma_{l2}^{2} \end{array}\right]$ for the litter effect, and $\left[\begin{array}{cc} I_{\varepsilon 1}⊗\sigma_{\varepsilon 1}^{2} & 0\\ 0 & I_{\varepsilon 2}⊗\sigma_{\varepsilon 2}^{2} \end{array}\right]$ for the residual effects, and where ***I ***are identity matrices of appropriate size.

The first seven models (mod(1) to mod(7)) assumed no multiplicative coefficient for the maternal genetic effect, regardless of the number of lambs reared, that is $\alpha_{1}=\alpha_{2}=1$. The corresponding tested models differed at the parameter level, the latter being estimated in the covariance matrices (Table 2). Mod(1) corresponded to the classical single trait model: regardless of the number of lambs reared, the direct (maternal) genetic effects ($\sigma_{d1}^{2}=\sigma_{d2}^{2},\sigma_{d1d2}^{}=\sigma_{d1}^{}\sigma_{d2}^{};\sigma_{m1}^{2}=\sigma_{m2}^{2},\sigma_{m1m2}^{}=\sigma_{m1}^{}\sigma_{m2}^{}$) and the maternal permanent effects ($\sigma_{p1}^{2}=\sigma_{p2}^{2},\sigma_{p}^{}=\sigma_{p1}^{}\sigma_{p2}^{}$) were identical, and the variance of the litter effect ($\sigma_{l1}^{2}=\sigma_{l2}^{2}$) and the residual variance ($\sigma_{\varepsilon 1}^{2}=\sigma_{\varepsilon 2}^{2}$,) did not vary. Mod(2) assumed that the maternal permanent effect depended on the number of lambs reared. Mod(3) allowed the residual variance to differ between single- and twin-reared lambs. It should be noted to allow for identifiability, mod(3) (and, for the same reason, mod(4) to mod(7)) considered no litter effect for observations on single-reared lambs; i.e. $\sigma_{l1}^{2}=0$. Mod(4) assumed that both the maternal permanent effect and residual variance depended on the number of lambs reared. Mod(5) (mod(6)) assumed, in addition, that the direct (maternal) genetic effect differed between single and twin-lambs. Finally, mod(7) corresponded to the global model, in which all parameters were estimated (except $\sigma_{l1}^{2}\;$). The last model (mod(coef)) was derived from the French indexation method of accounting for the heterogeneity between single- and twin- reared lambs. Mod(coef) made the same assumptions as mod(1) but considered, in addition, a multiplicative coefficient for the maternal genetic effect, i.e. $\alpha_{1}=1,\alpha_{2}=0.7$.

**Table 2:** Assumptions of the different models

		Direct genetic			Maternal genetic			Maternal permanent			Litter		Residual	
	*α*_2_	$\sigma_{d_{1}}^{2}$	$\sigma_{d_{2}}^{2}$	$\rho_{d_{1}d_{2}}^{}$	$\sigma_{m_{1}}^{2}$	$\sigma_{m_{2}}^{2}$	$\rho_{m_{1}m_{2}}^{}$	$\sigma_{p_{1}}^{2}$	$\sigma_{p_{2}}^{2}$	$\rho_{p_{1}p_{2}}^{}$	$\sigma_{l_{1}}^{2}$	$\sigma_{l_{2}}^{2}$	$\sigma_{e_{1}}^{2}$	$\sigma_{e_{2}}^{2}$

Mod(7)	= 1	✓	✓	✓	✓	✓	✓	✓	✓	✓	✓		✓	✓
Mod(6)	= 1	✓	✓	✓	✓		= 1	✓	✓	✓	✓		✓	✓
Mod(5)	= 1	✓		= 1	✓	✓	✓	✓	✓	✓	✓		✓	✓
Mod(4)	= 1	✓		= 1	✓		= 1	✓	✓	✓	✓		✓	✓
Mod(3)	= 1	✓		= 1	✓		= 1	✓		= 1	✓		✓	✓
Mod(2)	= 1	✓		= 1	✓		= 1	✓	✓	✓	✓		✓	
Mod(1)	= 1	✓		= 1	✓		= 1	✓		= 1	✓		✓	
Mod(Coef)	= 0.7	✓		= 1	✓		= 1	✓		= 1	✓		✓	

✓ in two cells indicates that the two components are equal; = × indicates that the component is fixed to x. for litter size i; $\sigma_{e_{i}}^{2}$ is the residual variance; $\sigma_{d_{i}}^{2}$ and $\rho_{d_{1}d_{2}}^{}$ are the direct genetic variance and correlation; $\sigma_{m_{i}}^{2}$ and $\rho_{m_{1}m_{2}}^{}$ are the maternal genetic variance and correlation; $\sigma_{p_{i}}^{2}$ and $\rho_{p_{1}p_{2}}^{}$ are the maternal permanent variance and correlation; $\sigma_{l_{i}}^{2}$ is the litter variance

All the fixed effects and one-way interactions of biological relevance included in the models were selected beforehand in a step-wise manner, using nested models that were compared with the likelihood ratio test (including interactions with rearing type). The following effects were tested: type of birth, sex of the lamb, year, season, age of the dam, age of the sire, and age of the lamb at weighing. Models were fitted using the mixed procedure of SAS^® ^8.1 (SAS^®^, version 8, 1999). After removal of non-significant effects, the following combinations of effects were retained: type of birth*sex of the lamb, year*season, and age of the dam for each rearing type.

All models were fitted using Asreml software [13]. Estimates of heritability was computed based on resulting estimates of variance and co-variance components, based on $\alpha_{i}^{2}\sigma_{mi}^{2}/)\left(\alpha_{i}^{2}\sigma_{mi}^{2}+\sigma_{di}^{2}+\alpha_{i}^{}\sigma_{dimi}+\sigma_{pi}^{2}+\sigma_{li}^{2}+\sigma_{\varepsilon i}^{2}\right)$ for the maternal effect and $\sigma_{di}^{2}/)\left(\alpha_{i}^{2}\sigma_{mi}^{2}+\sigma_{di}^{2}+\alpha_{i}^{}\sigma_{dimi}+\sigma_{pi}^{2}+\sigma_{li}^{2}+\sigma_{\varepsilon i}^{2}\right)$ for the direct effect. Models were compared using the Akaike information criteria (AIC).

Once the most parsimonious model which best fitted the data had been identified, the estimated EBV were compared to those obtained with mod(coef). Furthermore, the stability of EBV estimations for females having reared single and then twin lambs was compared for mod(coef) and the model which best fitted the data by reanalyzing two data subgroups: data1 included all records prior to 2005 (23,521 records, 5,214 dams) and data2 included all records prior to 2006 (25,385 records, 5,590 dams). The year 2005 was selected as a cut-off date because it ensured us with a maximal number of "selected" females (43), i.e. females that reared twin lambs for the first time in 2006 after having reared single lambs at least twice before. We then investigated, for all two methods, whether the selected females showed a reduced EBV when compared to the group "all females". For these comparisons, we 1) compared maternal EBV obtained with data1 and data2, 2) performed the Wilcoxon rank sum test to compare the distribution of rank between "selected" and all other females (i.e. all females excluding selected females), and 3) compared the number of "selected" females in each quartile of the EBV distribution in 2005 and 2006 based on the Chi-square statistic of the 2 × 4 contingency table.

## Results

The variance components and AIC obtained with the different models are presented in Table 3. A comparison of the different models shows that both the direct effects and maternal genetic effects were the same for single and twin lambs (AIC between mod(7) and mod(5) or mod(6) and mod(4) for direct effects, and between mod(7) and mod(6) or mod(5) and mod(4) for maternal effects). The maternal permanent effect differed between single and twin lambs (comparison of mod(4) with mod(3)). Heterogeneity was observed between the residual variances for single and twin lambs (comparison of mod(2) with mod(4)). Mod(4) shows the lowest AIC. This model assumed heterogeneity of residual variances and that the dam permanent effect differed between single and twin lambs.

**Table 3:** Estimates of variance components, heritabilities (s.e.), correlations (s.e.) and AIC obtained with the different models

	Mod(Coef)	Mod(1)	Mod(2)	Mod(3)	Mod(4)	Mod(5)	Mod(6)	Mod(7)
$\sigma_{e_{1}}^{2}$ $\sigma_{e_{2}}^{2}$	1581.87	1688.97	1633.56	2260.68 1556.34	2085.98 1556.18	2086.10 1556.70	2073.32 1563.27	2033.06 1566.96
$\sigma_{d_{1}}^{2}$ $\sigma_{d_{2}}^{2}$	390.34	416.33	403.72	384.93	385.85	385.04	415.35 422.07	473.15 366.79
$\sigma_{m_{1}}^{2}$ $\sigma_{m_{2}}^{2}$	347.11	284.58	180.16	181.83	179.71	228.20 198.44	179.52	265.40 168.52
$\sigma_{p_{1}}^{2}$ $\sigma_{p_{2}}^{2}$	219.50	225.86	719.60 211.30	232.50	454.17 212.17	419.12 219.31	441.14 215.56	416.21 218.47
$\sigma_{l_{2}}^{2}$	355.99	202.93	275.24	315.35	323.78	323.97	324.74	325.07

$h_{d_{1}}^{2}$	0.13 (0.01)	0.16 (0.02)	0.14 (0.01)	0.13 (0.01)	0.12 (0.01)	0.12 (0.01)	0.13 (0.03)	0.15 (0.03)
$h_{m_{1}}^{2}$	0.12 (0.02)	0.11 (0.01)	0.06 (0.01)	0.06 (0.01)	0.06 (0.01)	0.07 (0.02)	0.06 (0.01)	0.08 (0.02)
$h_{d_{2}}^{2}$	0.14 (0.01)	0.15 (0.02)	0.15 (0.02)	0.14 (0.02)	0.14 (0.02)	0.14 (0.02)	0.14 (0.02)	0.14 (0.02)
$h_{m_{2}}^{2}$	0.06 (<0.01)	0.10 (0.01)	0.06 (0.01)	0.07 (0.01)	0.07 (0.01)	0.06 (0.01)	0.07 (0.01)	0.06 (0.01)

$\rho_{d_{1}d_{2}}^{}$							1.00 (0.06)	1.00 (0.09)
$\rho_{m_{1}m_{2}}^{}$								0.89 (0.14)
$\rho_{d_{1}m_{1}}^{}$	0.08 (0.09)	0.11 (0.09)	0.05 (0.09)	0.07 (0.10)	0.07 (0.10)	0.07 (0.13)	0.13 (0.14)	-0.10 (0.19)
$\rho_{d_{2}m_{2}}^{}$								0.10 (0.11)
$\rho_{d_{1}m_{2}}^{}$						0.09 (0.11)		0.13 (0.16)
$\rho_{d_{2}m_{1}}^{}$							0.06 (0.10)	0.00 (0.14)
$\rho_{p_{1}p_{2}}^{}$			0.60 (0.06)		0.76 (0.09)	0.73 (0.11)	0.73 (0.09)	0.74 (0.11)

AIC	322	486	354	300	288	294	292	298

For litter size i, $\sigma_{e_{i}}^{2}$ is residual variance; $\sigma_{d_{i}}^{2}$ direct genetic variance; $\sigma_{m_{i}}^{2}$ maternal genetic variance; $\sigma_{p_{i}}^{2}$ maternal permanent variance; $\sigma_{l_{i}}^{2}$ litter variance; $h_{d_{i}}^{2}$ heritability for direct effect; $h_{m_{i}}^{2}$ heritability for maternal effect; $\rho_{d_{i}m_{j}}^{}$ correlation between direct (i) and maternal (j) effects; $\rho_{d_{1}d_{2}}^{}$ correlation between direct genetic effects; $\rho_{m_{1}m_{2}}^{}$ correlation between maternal genetic effects; $\rho_{p_{1}p_{2}}^{}$ correlation between maternal permanent effects. Figures across two lines indicate that the two components are equal.

Estimates of heritabilities obtained with the different models were consistent (Table 3). The heritability of the direct effect was moderate and ranged from 0.12 to 0.16 for single-reared lambs and from 0.14 to 0.15 for twin-reared lambs, depending on the model. The heritabilities obtained for maternal effects were low for all models and ranged from 0.06 to 0.12 for single-reared lambs and from 0.05 to 0.10 for twin-reared lambs. The genetic correlation between direct and maternal effects was low and did not differ from 0 in all models.

When the maternal permanent effect was considered to be different for single- and twin-reared lambs (mod(2) and mod(4) to mod(7)), the variance of the permanent effect of dams was higher for single-reared lambs (ranging from 416.21 to 719.60 depending on the model) than for twin-reared lambs (ranging from 211.30 to 219.31, depending on the model). The correlation between the two permanent effects was generally high, ranging from 0.60 to 0.76 depending on the model, but different from 1 (AIC between mod(4) and mod(3), between mod(2) and mod(1)). The results were consistent for the different models that assumed heterogeneous residual variances (mod(3) to mod(7)). The residual variance was higher for single-reared lambs (1.1 to 1.4 fold) than for twin-reared lambs. Litter variance represented 7 to 12% of the total variance, depending on the model.

Correlations between the EBV obtained with the model showing the lowest AIC (mod(4)) and mod(coef) are presented in Table 4. Correlations were high: 0.979 for maternal effects and 0.998 for direct effects. The percentage of animals in common among animals with the 10% highest or the 10% lowest EBV for the two models was high for the direct effect (93 and 96%) and slightly lower for the maternal effect (79%).

**Table 4:** Agreement between EBV estimated with the model that best fitted the data (mod(4)) and with mod(Coef)

		Direct effect	Maternal effect
Correlation between EBV		0.998	0.979
Percentage of animals in common among animals with the	10% best EBV	93	79
	10% worst EBV	96	79

In order to determine whether the maternal EBV of ewes that previously reared single-suckling lambs decreases when they subsequently rear two or more lambs ("selected" females), comparisons of EBV obtained in 2005 and 2006 with the model that best fitted the data (mod(4)) and mod(coef) based on the Wilcoxon rank sum test and the chi-square statistic are presented in Table 5. For both models, the mean EBV for selected females were not significantly different in 2005 and 2006 (p = 0.45 and p = 0.24 for mod(4) and mod(coef), respectively). None of the Wilcoxon rank-sum tests were significant, indicating that no differences could be observed in the position of the "selected" females in comparison to all females, regardless of the model or the year of evaluation. Finally, for both models, the chi-square statistic of the contingency table which compared the number of "selected" females in each quartile of the EBV distribution in 2005 and 2006 was not significant (p > 5%). All these results indicate no evidence of a decrease of the maternal EBV of ewes that rear twins for the first time after previously having reared only single lambs.

**Table 5:** Comparison of maternal EBV between selected and all females estimated with mod(Coef) and the model which best fitted the data (mod(4))

		Mod(Coef)		Mod(4)	
		**All animals^1^**	**Selected females**^**2**^	**All animals^1^**	**Selected** **females^2^**

Mean EBV (std)	Data1 Data2	8.4 (9.4) 8.5 (9.9)	9.4 (9.0) 8.4 (9.5)	6.3 (7.1) 6.2 (7.3)	6.5 (5.5) 6.6 (6.6)
Wilcoxon rank-sum test^3^	Data1 Data2	0.23 0.29		0.27 0.36	
$\chi_{3ddl}^{2}$ test^4^		0.84		0.82	

^1^756 females having records in 2005 and 2006; ^2^43 females having twin lambs for the first time in 2006 after having reared single lambs at least twice; ^3^p value of the wilcoxon rank-sum test to test if the distributions of rank of all versus selected females are different; ^4^p value of the chi-square test to test if the percentages of selected females in each quartile of the EBV distribution are different in 2005 and 2006; Data1: all records before 2005; Data2: all records before 2006

## Discussion

The data we used came from an experimental farm, which provides some advantages over field data. For instance, weight recordings were performed in a standardized manner; weight at birth was measured within 12 h after lambing and weight at day 45 was measured very close to the actual 45^th ^day of life. This avoided approximations by interpolation in the calculation of the ADG. However, the use of such experimental data has the disadvantage of including relatively few records and special attention must be paid to make sure that the data can disentangle direct and maternal effects. In this particular dataset, we are confident that this is the case for single trait analyses (mod(1)) because of the strong genetic relationships between individuals, especially cousin relationships. The mean number of records per dam, sire and maternal granddam for single reared-lambs was low (1.5, 6.1 and 8.6, respectively). However, these animals were also parents of twin reared-lambs. Consequently, records from twins provided the necessary information to estimate random parameters for single reared-lambs (if correlated) and helped to disentangle the direct and maternal effects for single reared-lambs when estimated in the case of multiple-trait assumptions. This was confirmed by the consistency of the estimates of heritabilities and correlations between models.

We decided to analyze the hypothetical differences between single- and twin-reared lambs by testing for differences between singles and twins for all random components of the model. At present, the results reported in the literature are in favour of a difference between the effects associated with singles and twins. Concerning direct effects, it has been reported that the behaviour of single-reared lambs is different from that of twin-reared lambs. On pasture, single-reared lambs were usually further from their dams than were multiple-reared lambs [7]. It has also been shown that single lambs suckled less frequently but longer than twins [7,14]. In other species, it has been reported that the behavioural mechanisms of sibling competition range from very aggressive interactions, through various milder agonistic interactions, to scramble competition [7]. Although, to our knowledge, such mechanisms have not been reported in sheep, we can assume that competitive behaviour also exists in this species. With regards to maternal effects, the lactation curve differs between ewes nursing single and twin lambs. Ewes suckling twins have been shown to produce more milk than those suckling single lambs; their peak yield is reached during the 3^rd ^week of lactation, compared with the 4^th ^week for ewes with single lambs, and they show higher persistency [3,5]. Furthermore, ewes with twins have higher milk fat levels and produce more milk energy than those with single lambs [15]. From a genetic point of view, these differences could be interpreted as differences in both the ewe's and lamb's environmental conditions depending on the number of lambs reared. However, the results we obtained did not support the hypothesis of a genetic by environment interaction between single and twin lambs, which we evaluated with a multiple-trait model; the genetic correlation between the direct (maternal) effects for single or twin lambs was not significantly different from 1 and their variances did not differ. These results are not consistent with those obtained by Buvanendran et al. [16], who reported that genetic variance and heritability were greater for twins, although heritabilities were not significantly different.

Our results demonstrate that the maternal permanent effect was not the same when ewes reared single versus twin lambs. The permanent effect of dam accounts for all environmental factors related to the dam that are not explicitly incorporated in the model but which modify the non-genetic component of the maternal environment and therefore influence the growth of the lambs. A difference in permanent effects of dams for single versus twin lambs indicates that some of those unaccounted factors exert different effects depending on the number of lambs reared. One of these factors could be impairment of one quarter due to mastitis, which would have a negative influence on the ability of the ewe to rear two lambs but not on her ability to suckle a single lamb.

Our results for the relative importance of the litter effect (7 to 12%) are in the range of those reported in previous studies (0.11 [17]) or slightly lower (0.26 to 0.31 [18]). The litter effect is a combination of everything that affects members of a litter in the same way, including environmental conditions that are not accounted for by the other effects included in the model, and maternal temporary environmental effects (ewe*year effect in our case).

The results obtained here are in favour of different residual variance for single- versus twin-reared lambs. The raw data showed that single lambs have a higher ADG and a higher standard deviation than twins. The difference in variance was not due to a mean and variance relationship. In fact, the data were normally distributed and the slope of the regression linking the standard deviation of the raw data to the mean (with 10 g steps) was null (3.2.10^-4^).

Variances of dam permanent and residual effects were higher for single- than twin-reared lambs. One possible explanation for these differences is that, in the case of single-reared lambs, the observed ADG represents the "optimal" growth that can be obtained for the corresponding lamb-ewe-environment combination, while the competition between twin-reared lambs results in only part (*α*%) of this optimal growth to be expressed. In other words, if we only consider random factors: $y_{1.obs_{ij}}=y_{optimal_{ij}}=d_{i}+m_{j}+p_{j}+\varepsilon_{ij},y_{2.obs_{ij}}=\alpha y_{optimal_{ij}}$ where $y_{1.obs_{ij}},y_{2.obs_{ij}}$refer to the observed ADG for the single or twin lamb *i *of ewe *j*, respectively, and other notations are the same as for the general model. Under this assumption, the variances of all random factors for single lambs are higher than for twins and this is consistent with the results obtained in this study. In fact, although not significantly different from 1 for the genetic effects, the ratio between the variances of random factors for single and twin lambs varied from 0.7 to 0.9 for the different factors in mod(7). Although convenient, this hypothesis oversimplifies the problem because the correlation between the permanent effects of the dam is not equal to 1 between single- and twin-reared lambs.

Our estimates of heritability are consistent with most of the heritabilities reported in the literature for pre-weaning ADG in sheep. Bromley et al. [19] reported heritabilities varying from 0.07 to 0.20 for direct effects and from 0.04 to 0.05 for maternal effects, depending on the breed. In a review, Safari et al. [2] reported an average heritability of 0.15 for the direct effect and 0.05 for the maternal effect. Heritability was also higher for the direct effect (0.21) than for the maternal effect (0.01) in Mousa et al. [20]. Hagger [18], when comparing models in two breeds, obtained heritabilities varying from 0.08 to 0.16 for direct effects and from 0.02 to 0.10 for maternal effects. On the contrary, Snowder and Van Vleck [21] reported a low heritability for direct effects (0.03) and a higher heritability for maternal effects (0.28). Estimates of the genetic correlation between direct and maternal effects obtained in previous studies vary to a much greater extent, from -0.52 [20] to 0.52 [19]. Our close to 0 estimate of the genetic correlation is consistent with the review by Safari et al. (-0.02 (0.08)) [2]. It is a well-known fact that estimates of this correlation are particularly sensitive to data structure [22-24] but, as previously mentioned, working with experimental data from a single herd probably overcomes this bias. The genetic parameters used in the French genetic evaluation model are heritabilities of 0.20 for the direct effect and 0.30 for the maternal effect, and -0.4 for the genetic correlation, (J.P. Poivey, personal communication). The discrepancy between these parameters and those estimated in the present study indicates that it may be of interest to update the parameters for field data.

We did not find any spurious changes in the maternal EBV of ewes rearing twin lambs for the first time after having reared single lambs the previous years, as had been reported from the field. One explanation for this result is that problems reported from field data are due to the quality of the data recorded, especially absence of recording lamb deaths which introduces bias in the type of rearing factor. This problem does not exist for the experimental data used for this study.

In this study, we focused on the possible heterogeneity of variance components for pre-weaning growth in sheep due to the number of lambs reared in order to check if the multiplicative coefficient assumptions made in the French genetic evaluation system are valid. Several other factors have been reported in the literature to affect early growth but are not included at present in the French genetic evaluation model and can introduce biases. A non-exhaustive list of these factors is the following: an environmental covariance between dam and offspring [25,26], sire*year, sire*herd*year [23,27], sire*dam, dam*number born [28] combinations, etc. The importance of these factors should be tested on field data when updating the French genetic evaluation model.

## Conclusions

The objective of this study was to evaluate the best way to take account for differences in pre-weaning growth between single- and twin-reared lambs in comparison with the method used at present in the French genetic evaluation model. Our results show that the genetic effects do not differ between single- and twin-reared lambs, that the permanent environmental effect of dams depends on the number of lambs suckled, that the residual variance is different for single and twin lambs and that it is better to consider these assumptions than to apply a multiplicative coefficient to the maternal genetic effect. Given these results from experimental data, it would be of interest to compare a model that includes all these new assumptions with the model used at present for the genetic evaluation in other breeds with field data and update the genetic evaluation model based on the results obtained.

## Competing interests

The authors declare that they have no competing interests.

## Authors' contributions

ID performed statistical analysis and drafted the manuscript. DF performed data edition. FB was responsible for recording data. JPP and LT are responsible for the current genetic evaluation for pre-weaning growth. All authors have been involved in drafting the manuscript and proofing and have approved the final manuscript.
